# Effect of basal LH levels on pregnancy outcome after IVF/ICSI fresh embryo transfer in patients of different ages: a retrospective study

**DOI:** 10.3389/fendo.2026.1820385

**Published:** 2026-05-15

**Authors:** Mengying Zhang, Qixuan Zhang, Xiangyang Ou, Ying Zhang, Gengxiang Wu

**Affiliations:** 1 Center for Reproductive Medicine, People’s Hospital of Wuhan University, Wuhan, China; 2 Hubei Clinical Research Center for Reproductive Medicine, Shiyan, Hubei, China; 3 Shiyan Key Laboratory of Reproduction and Genetics, Renmin Hospital, Hubei University of Medicine, Shiyan, Hubei, China

**Keywords:** age, embryo transfer, IVF, luteinizing hormone, pregnancy outcome

## Abstract

**Background:**

The predictive value of basal luteinising hormone (LH) levels for pregnancy outcomes following *in vitro* fertilization/intracytoplasmic sperm injection (IVF/ICSI) remains controversial, and whether this relationship differs by female age has not been fully established.

**Methods:**

A total of 1153 patients undergoing their first IVF/ICSI cycle with fresh embryo transfer at a university-affiliated reproductive medicine center between March 2020 and May 2022 were retrospectively analyzed. Patients were stratified by basal LH quartiles (<25th, 25th-75th, >75th) and age groups (<35, 35-37, and ≥38 years). Multivariable logistic regression was used to adjust for confounders, with candidate variables selected based on univariate analysis (P < 0.10) and clinical relevance. The primary outcomes were clinical pregnancy rate (CPR) and live birth rate (LBR).

**Results:**

The CPR and LBR declined progressively with increasing age (both P < 0.001). In patients aged <35 years, the >75th basal LH quartile was significantly associated with higher CPR (adjusted OR 1.885, 95% CI 1.267-2.803, P = 0.002) and higher LBR ((adjusted OR 1.702, 95% CI 1.146-2.529, P = 0.008) compared with the <25th quartile, after adjusting for age, body mass index, infertility cause, ovarian reserve markers, stimulation protocol, and embryo quality. Progesterone level on the day of human chorionic gonadotropin administration was negatively associated with CPR in this age group ((adjusted OR 0.603, 95% CI 0.402-0.906, P = 0.015). In patients aged 35–37 years, endometrial thickness was positively associated with both CPR and LBR, whereas no significant association between basal LH and pregnancy outcomes was observed. In patients aged ≥38 years, no significant association was found between basal LH and either CPR or LBR, and confidence intervals for basal LH estimates were notably wide in this subgroup.

**Conclusion:**

Elevated basal serum LH levels are associated with higher CPR and LBR following fresh embryo transfer in patients aged <35 years, but this association is not observed in women aged ≥35 years.

## 1 Introduction

In recent years, advances in assisted reproductive technologies have led to increased adoption of *in vitro* fertilization/intracytoplasmic sperm injection-embryo transfer (IVF/ICSI-ET) among infertility patients. Controlled ovarian hyperstimulation (COH) has become a central component of this treatment approach. During COH, to obtain an appropriate number of mature oocytes, it is essential to thoroughly assess ovarian reserve function and responsiveness. Numerous studies indicate that age, basal luteinising hormone (LH) levels, and basal follicle-stimulating hormone (FSH) levels are crucial indicators for evaluating ovarian responsiveness (1). Research indicates endogenous LH activity is essential for optimal follicular development, with low LH levels potentially signaling impaired hypothalamic-pituitary-ovarian axis function (2). LH levels are also known to correlate closely with age. Normal follicular growth and maturation require combined stimulation from FSH and LH, though FSH is considered predominant (3). Research on whether basal LH levels correlate with pregnancy outcomes following fresh embryo transfer remains scarce and controversial (4). This retrospective study therefore aims to analyze the impact of basal LH levels across different age groups on IVF/ICSI fresh embryo transfer outcomes.

## 2 Materials and methods

### 2.1 Study population

This retrospective analysis examined clinical data from 1799 patients undergoing IVF/ICSI with fresh embryo transfer at Wuhan University People’s Hospital between March 2020 and May 2022. Inclusion criteria were: (1) age 20–45 years; (2) Undergoing their first IVF/ICSI treatment with fresh embryo transfer. Following exclusion criteria screening, 1153 patients were ultimately included.

**Figure 1:**
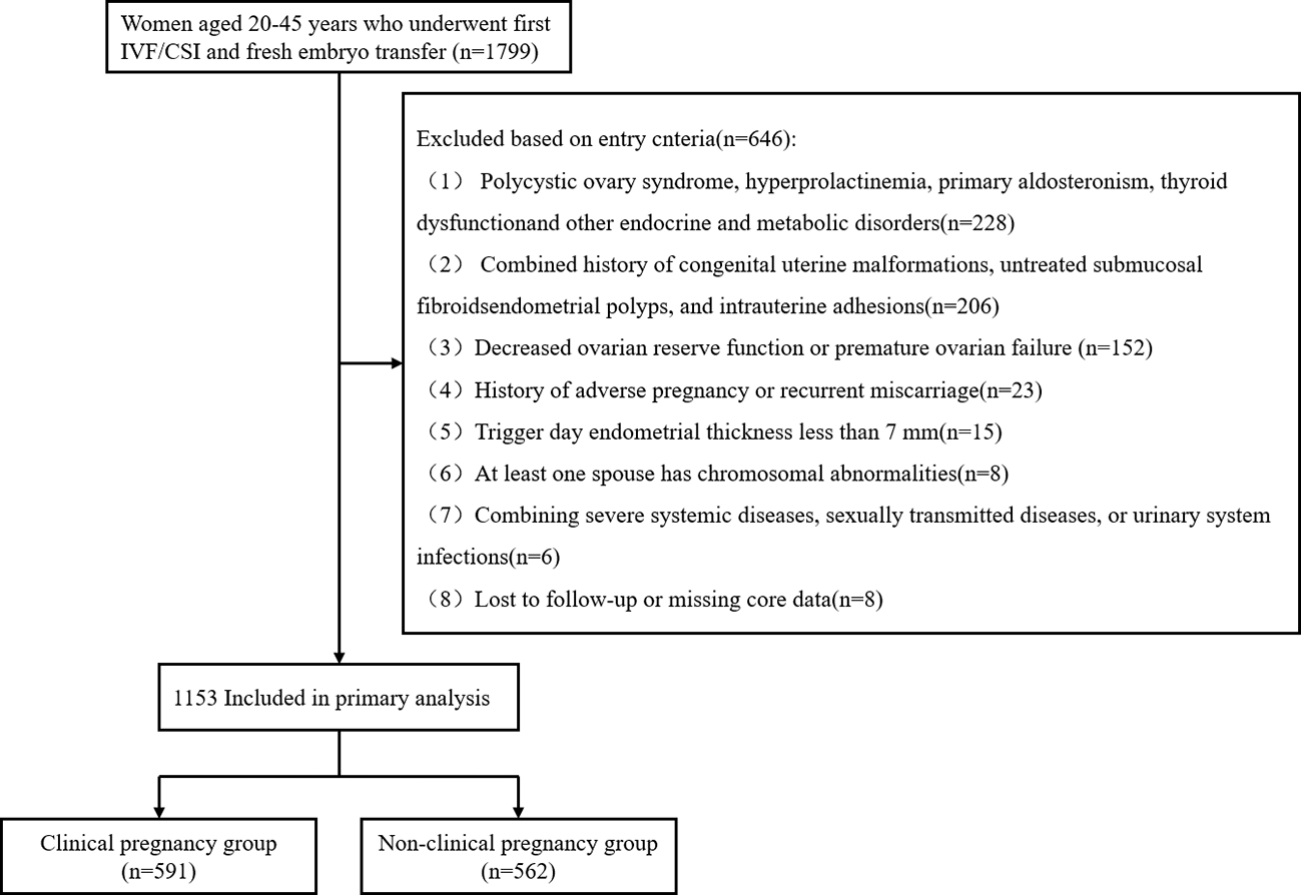
Flow chart for acceptance and discharge.

Specific exclusion criteria were: (1) Polycystic ovary syndrome and endocrine-metabolic disorders such as hyperprolactinemia, primary aldosteronism, or thyroid dysfunction; (2) Concurrent congenital uterine malformations, untreated submucosal fibroids, endometrial polyps, or history of intrauterine adhesions; (3) Declining ovarian reserve (defined as basal FSH >10 IU/L, or anti-Müllerian hormone (AMH) <1.1 ng/mL, or antral follicle count (AFC) <5–7) or premature ovarian failure (defined as amenorrhea for >4 months with FSH >40 IU/L on two occasions) (4); History of adverse pregnancy outcomes or recurrent miscarriage; (5) Endometrial thickness <7mm on trigger day; (6) Chromosomal abnormalities in either partner; (7) Concurrent severe systemic diseases, sexually transmitted infections, or urinary tract infections; (8) Loss to follow-up or missing core data (**Figure 1**).

This study was approved by the Ethics Committee of Renmin Hospital of Wuhan University [Ethics Approval No.: WDRM2024-K152] and was conducted in accordance with the 1964 Helsinki Declaration and its later amendments or comparable ethical standards. Informed consent was waived by our Institutional Review Board because of the retrospective nature of our study.

Participants were categorized into clinical pregnancy and non-clinical pregnancy groups based on clinical pregnancy occurrence, with comparative analysis of clinical characteristics. Further stratification by basal LH quartiles (<25th, 25th-75th, >75th) was conducted to evaluate clinical outcome indicators during fresh embryo transfer cycles. Finally, subgroup analyses were conducted for patients in different age groups: <35years, 35–37 years, and ≥38years (5–8), comparing pregnancy outcomes across these age groups for patients with varying basal LH levels. To ensure consistency, all basal hormone measurements were performed on day 2–3 of the menstrual cycle before gonadotropin administration, under standardized laboratory conditions.

### 2.2 Ovulation induction protocols

Common protocols include the antagonist protocol, long protocol, and ultra-long protocol. (1) Antagonist Protocol: Patients commence recombinant follicle-stimulating hormone (rFSH, Merck Serono, Germany) or human menopausal gonadotropin (HMG, Zhuhai Lizu, China) at 150–300 U/day on day 2 or 3 of the menstrual cycle. Gonadotropin dosage is adjusted based on serum hormone levels and follicular development assessed by ultrasound. When dominant follicles reach 12–14 mm in diameter, administer GnRH antagonist (Triptorelin, Merck, Switzerland) at 0.25 mg/day. When at least two dominant follicles exceed 18mm in diameter or three dominant follicles measure ≥17 mm, administer human chorionic gonadotropin (hCG, Zhuhai Lizu, China) 6000–10000 U to induce ovulation. Perform transvaginal ultrasound-guided follicular aspiration 34–36 hours later. (2) Long Protocol: Initiate daily GnRH-a injections (Diphereline, Ferring, Germany) at 0.1 mg during the mid-luteal phase. After 14 days, assess serum hormone levels and perform ultrasound evaluation. If downregulation criteria are met (E2<50pg/ml, LH ≤3IU/L, follicle diameter <10mm), initiate daily Gn (FSH or HMG) injections at 150-300U/day, with dosage adjusted according to ovarian reserve function. Trigger criteria and methodology are identical to the antagonist protocol. (3) Ultra-long protocol: Initiate long-acting GnRH-a (3.75mg, Decapeptyl, Ipsen, France) on day 2 of menstruation. Reassess serum hormone levels and perform ultrasound scan after 28 days. Upon achieving target suppression, commence ovarian stimulation. Subsequent Gn administration, follicular monitoring, trigger criteria, and methods are identical to the antagonist protocol.

### 2.3 Oocyte retrieval, *in vitro* fertilization and embryo transfer

Oocytes are retrieved via transvaginal ultrasound-guided aspiration from follicles ≥10mm in diameter. Following retrieval, conventional IVF or ICSI is performed based on male semen parameters. Ovum retrieval was performed 34–36 hours after HCG trigger under vaginal ultrasound guidance, sequentially aspirating follicles exceeding 10mm in diameter. The choice between IVF or ICSI was determined based on male semen parameters and fertilization outcomes from previous cycles. Fertilization status was assessed 18 hours post-retrieval, with embryo evaluation conducted at 48 and 72 hours post-retrieval. Embryos are graded into four categories based on blastomere size, morphology, and fragmentation rate: Grade I: Uniform blastomere size, regular morphology with clear cytoplasm, no fragmentation or fragmentation rate <10%; Grade II: Variable blastomere size, irregular morphology, fragmentation rate 10%-26%; Grade III: Unevenly sized blastomeres with a fragmentation rate of 26%-50%; Grade IV: Severely unevenly sized blastomeres with a fragmentation rate exceeding 50%. Embryos graded as Grade I or Grade II at 72 hours post-retrieval indicate favorable quality and are defined as high-quality embryos. Fresh embryo transfer is performed under abdominal ultrasound guidance 3–5 days post-retrieval, transferring 1–2 embryos.

### 2.4 Luteal support therapy and clinical outcome follow-up

Luteal support therapy commences on the day following egg retrieval, involving daily injections of 40 mg progesterone (Zhejiang Xianju Pharmaceutical). Oral dydrogesterone tablets (Abbott, Netherlands) at 30 mg daily are initiated on the day of embryo transfer. Blood β-HCG levels were measured 10–12 days post-embryo transfer. β-HCG level >10 U/L was considered HCG-positive, warranting continued luteal support. Transvaginal ultrasound was performed 28–30 days post-transfer; clinical pregnancy was defined as the presence of a gestational sac with primitive cardiac tube activity. Luteal support continued until 10 weeks of gestation, with follow-up extending to approximately 12 weeks.

### 2.5 Observation indicators

Primary pregnancy outcome indicators were clinical pregnancy rate (CPR) and live birth rate (LBR). Additional pregnancy outcome indicators included HCG positivity rate and embryo implantation rate.

### 2.6 Statistical methods

Analysis was performed using SPSS 26.0 statistical software. Normally distributed continuous variables are expressed as mean ± standard deviation $\left(\bar{x}\pm s\right)$, while skewed continuous variables are presented as median (interquartile range) [M(IQR)]. Intergroup comparisons employed t-tests or Mann-Whitney *U* nonparametric tests. Categorical variables were expressed as frequency (n) and percentage (%). Intergroup comparisons employed the chi-square test. Logistic regression analysis was conducted, incorporating variables demonstrating intergroup differences in univariate regression and clinically relevant variables into a multivariate model. For multivariable logistic regression analyses, variables were selected based on univariable logistic regression for clinical pregnancy rate within each age group (**Supplementary Table 1**), with those showing P < 0.10 being considered as candidates, together with clinically relevant variables (such as infertility cause, stimulation protocol, and number of high-quality embryos), for inclusion in the multivariable model. After adjusting for confounding factors, this explored factors influencing pregnancy outcomes following fresh embryo transfer. Collinearity among independent variables was assessed using variance inflation factor (VIF) and tolerance values. All VIF values were below 5 and all tolerance values were above 0.1, indicating no significant multicollinearity in the multivariable models (**Supplementary Table 2**). P value < 0.05 was considered statistically significant.

## 3 Result

### 3.1 Comparison of clinical characteristics between the clinical pregnancy group and non-clinical pregnancy group

As shown in **Table 1**, the clinical pregnancy group had significantly lower age, basal E_2_, LH and P on hCG day, and higher basal LH, AMH, AFC, and endometrial thickness (all P < 0.05). Gn starting dose was lower in the pregnancy group (P < 0.05). No significant differences were observed in BMI, infertility duration, basal FSH, infertility type, total Gn dose, Gn duration, or embryo laboratory parameters (P > 0.05).

**Table 1:** Comparison of clinical characteristics between the two groups of patients [*M (IQR),%*].

Item	Clinical pregnancy group	Non-clinical pregnancy group	*Z*/ $x^{2}$value	*P* value
No. of cases	591	562		
Age (year)	31.0 (4.0)	32.0 (6.0)	-3.23	0.001
Infertility duration (year)	3.0 (2.0)	3.0 (2.0)	-0.32	0.752
BMI (kg/m^2^)	21.8 (4.1)	21.8 (3.9)	-0.52	0.604
Basal E_2_ (pg/mL)	40.2 (19.6)	43.6 (22.2)	-2.77	0.006
Basal LH (U/L)	3.8 (2.2)	3.6 (1.9)	-2.04	0.041
Basal FSH (U/L)	7.8 (2.2)	7.7 (2.6)	-0.27	0.789
Basal AMH (ng/mL)	2.7 (2.2)	2.5 (1.9)	-3.18	0.001
AFC	15 (9)	13 (8)	-3.38	0.001
Type of infertility (%)			2.23	0.136
Primary infertility	56.5 (334/591)	52.1 (293/562)		
Secondary infertility	43.5 (257/591)	47.9 (269/562)		
Ovulation protocol (%)			12.30	0.002
Antagonist protocol	21.0 (124/591)	29.7 (167/562)		
Long protocol	36.7 (217/591)	35.4 (199/562)		
Ultra-long protocol	42.3 (250/591)	34.9 (196/562)		
Gn starting dosage (IU)	187.5 (75.0)	200.0 (75.0)	-3.01	0.003
Total dosage of Gn used (IU)	2100.0 (836.3)	2125.0 (807.5)	-1.50	0.135
Duration of Gn used	10.0 (3.0)	10.0 (3.0)	-0.13	0.895
E_2_ on hCG injection day (pg/mL)	2525.0 (1409.9)	2546.0 (1608.8)	-0.32	0.753
LH on hCG injection day (U/L)	1.1 (1.3)	1.2 (1.5)	-2.50	0.012
P on hCG injection day (ng/mL)	0.9 (0.4)	0.9 (0.4)	-2.18	0.029
Endometrial thickness (mm)	12.0 (3.3)	11.6 (3.3)	-2.12	0.034
No. of oocytes retrieved	11.0 (7.0)	11.0 (7.0)	-1.76	0.078
No. of MII oocytes	8.0 (6.0)	8.0 (6.0)	-1.53	0.126
2PN cleavage rate (%)	98.0 (3766/3843)	98.2 (3397/3458)	0.56	0.453
Available embryo rate (%)	70.0 (2637/3766)	68.3 (2321/3397)	2.41	0.120

### 3.2 Comparison of clinical outcome indicators among groups following fresh embryo transfer cycles stratified by basal LH levels

**Table 2** presents clinical outcomes stratified by basal LH quartiles (<25th, 25th–75th, >75th). The >75th group exhibited significantly higher HCG positivity rate, clinical pregnancy rate, implantation rate, and live birth rate compared with the lower quartile groups (all *P* < 0.05). No significant differences were found in 2PN cleavage rate, high-quality embryo rate, or available embryo rate among the three groups (*P* > 0.05).

**Table 2:** Comparison of clinical outcome indicators of fresh embryo transfer cycles by groups stratified according to basal LH (%).

Item	Basic LH level stratified by percentile			$x^{2}$value	*P* value
	< 25th	25th–75th	> 75th		
No. of cases	288	578	287		
2PN cleavage rate (%)	98.3 (1787/1818)	98.2 (3554/3618)	97.7 (1822/1865)	2.36	0.308
High-quality embryo rate (%)	56.7 (1013/1787)	57.0 (2027/3554)	55.4 (1009/1822)	1.37	0.504
Available embryo rate (%)	67.0 (1197/1787)	70.0 (2488/3554)	69.9 (1273/1822)	5.58	0.061
HCG positive rate (%)	57.3 (165/288)	58.5 (338/578)	69.7 (200/287)^a,b^	12.31	0.002
Clinical pregnancy rate (%)	47.2 (136/288)	49.8 (288/578)	58.2 (167/287)^a.b^	7.87	0.019
Implantation rate (%)	33.5 (165/493)	37.7 (367/973)	44.1 (212/481)^a,b^	11.80	0.003
Live birth rate (%)	37.5 (108/288)	38.6 (223/578)	46.7 (134/287)^a,b^	6.52	0.038

^a,b^ Different superscripts within the same line means statistically difference between. <25th indicates basal LH level< 2.83 U/L;25th-75th indicates basal LH level of 2.83-4.91 U/L and>75th indicates basal LH level>4.91 U/L.

### 3.3 Comparison of clinical characteristics and pregnancy outcomes following stratification by basal LH levels across different age groups

As shown in **Table 3**, HCG positivity rate, clinical pregnancy rate, implantation rate, and live birth rate all declined significantly with increasing age (all *P* < 0.001).

**Table 3:** Comparison of pregnancy outcomes in patients of different ages (%).

Item	<35 years old	35–37 years old	≥38 years old	$x^{2}$value	*P* value
No. of cases	894	149	110		
HCG positive rate (%)	64.3 (575/894)	55.0 (82/149) ^a^	41.8 (46/110) ^a,b^	23.37	<0.001
Clinical pregnancy rate (%)	54.3 (485/894)	43.6 (65/149) ^a^	37.3 (41/110) ^a^	15.29	<0.001
Implantation rate (%)	41.2 (620/1505)	31.5 (78/248) ^a^	23.7 (46/194) ^a^	27.75	<0.001
Live birth rate (%)	44.2 (395/894)	30.2 (65/149) ^a^	22.7 (25/110) ^a^	26.03	<0.001

^a,b^ Different superscripts within the same line means statistically difference between subgroups.

**Supplementary Tables 3**–**5** present baseline characteristics by LH strata within each age group. **Table 4** details pregnancy outcomes for each age group stratified by basal LH levels. In patients aged <35 years, the <25th LH group had significantly lower HCG positivity rate, clinical pregnancy rate, implantation rate, and live birth rate compared with the >75th LH group (*P* < 0.05). No significant differences across LH strata were observed in patients aged 35–37 years or ≥38 years (*P* > 0.05).

**Table 4:** Comparison of pregnancy outcomes across basal LH strata in patients aged <35 years, 35–37 years, and ≥38 years.

Item	HCG positive rate	Clinical pregnancy rate	Implantation rate	Live birth rate
<35 years old				
<25th	56.5 (126/223)	45.7 (102/223)	33.0 (126/382)	37.7 (84/223)
25th–75th	63.9 (287/449)	54.8 (246/449) ^a^	42.5 (318/749) ^a^	44.1 (198/449)
>75th	73.0 (162/222) ^a,b^	61.7 (137/222) ^a^	47.1 (176/374) ^a^	50.9 (113/222) ^a^
*P* value	0.001	0.003	<0.001	0.019
35–37 years old				
<25th	65.8 (25/38)	52.6 (20/38)	35.8 (24/67)	36.8 (14/38)
25th–75th	47.3 (35/74)	37.8 (28/74)	28.1 (34/121)	27 (20/74)
>75th	59.5 (22/37)	45.9 (17/37)	33.3 (20/60)	29.7 (11/37)
*P* value	0.145	0.31	0.516	0.562
≥38 years old				
<25th	40.7 (11/27)	37.0 (10/27)	23.9 (11/46)	25.9 (7/27)
25th–75th	39.3 (22/56)	33.9 (19/56)	22.0 (22/100)	23.2 (13/56)
>75th	48.1 (13/27)	44.4 (12/27)	27.1 (13/48)	18.5 (5/27)
*P* value	0.739	0.65	0.793	0.804

^a,b^ Different superscripts within the same line means statistically difference between subgroups. The interquartile range (25th-75th) for LH in patients <35 years of age was 2.79-4.96 U/L; in patients 35–37 years of age it was 2.90-4.77 U/L; and in patients ≥38 years of age, it was 2.95-4.53 U/L.

### 3.4 Multivariate logistic regression analysis of factors influencing clinical pregnancy rates following fresh embryo transfer in patients of different ages

**Table 5** presents the multivariable logistic regression analysis for CPR. In patients aged <35 years, basal LH was significantly associated with CPR (P = 0.007). Compared with the <25th group, the >75th group had significantly higher CPR (adjusted OR = 1.885, 95% CI: 1.267-2.803, P = 0.002).Progesterone on hCG day was negatively associated with CPR (adjusted OR = 0.603, 95% CI: 0.402-0.906, P = 0.015). Other variables were not significantly associated with CPR in this age group.

**Table 5:** Multifactorial logistic regression analysis of clinical pregnancy after fresh embryo transfer in patients of different ages.

Item	<35 years old		35–37 years old		≥38 years old	
	*Adjusted OR (95%CI)*	*P*	*Adjusted OR (95%CI)*	*P*	*Adjusted OR (95%CI)*	*P*
Age	0.992 (0.944~1.043)	0.766	1.071 (0.684~1.678)	0.763	0.725 (0.542~0.970)	0.030
BMI≥24 kg/m^2^	1.125 (0.816~1.551)	0.471	2.437 (1.040~5.709)	0.040	1.721 (0.652~4.541)	0.273
Infertility factors		0.874				
Tubal factors	Reference		Reference	0.941	Reference	0.430
Male factors	0.888 (0.641~1.231)	0.476	0.976 (0.424~2.249)	0.955	1.851 (0.483~7.102)	0.369
Unexplained causes	0.886 (0.583~1.347)	0.570	0.678 (0.167~2.752)	0.587	1.321 (0.134~13.058)	0.812
Others	0.988 (0.625~1.563)	0.958	0.805 (0.235~2.763)	0.730	2.492 (0.835~7.438)	0.102
AFC	0.998 (0.975~1.022)	0.896	1.003 (0.938~1.072)	0.941	1.086 (0.961~1.227)	0.185
Basal AMH	1.041 (0.944~1.147)	0.418	0.849 (0.636~1.134)	0.269	1.087 (0.686~1.723)	0.721
Basal LH		0.007		0.454		0.066
<25*th*	Reference		Reference		Reference	
25*th*–75*th*	1.380 (0.988~1.927)	0.059	0.642 (0.253~1.624)	0.349	0.492 (0.155~1.560)	0.228
>75*th*	1.885 (1.267~2.803)	0.002	1.060 (0.373~3.008)	0.914	2.230 (0.608~8.170)	0.226
Treatment protocol		0.354		0.238		0.427
Antagonist protocol	Reference		Reference		Reference	
Long protocol	1.283 (0.888~1.854)	0.184	1.598 (0.620~4.117)	0.331	1.659 (0.563~4.885)	0.359
Ultra-long protocol	1.267 (0.873~1.839)	0.213	2.387 (0.872~6.531)	0.090	2.359 (0.599~9.285)	0.220
P on hCG injection day	0.603 (0.402~0.906)	0.015	0.896 (0.317~2.539)	0.837	0.236 (0.045~1.245)	0.089
Endometrial thickness on embryo transfer day	1.009 (0.958~1.063)	0.733	1.166 (1.025~1.326)	0.019	1.086 (0.872~1.352)	0.460
Number of high-quality embryos	1.031 (0.981~1.083)	0.233	1.107 (0.944~1.300)	0.211	1.078 (0.881~1.32)	0.464

The interquartile range (25th–75th) for LH in patients<35 years of age was 2.79-4.96 U/L; in patients 35–37 years of age,2.90-4.77 U/L; and in patients ≥38 years of age,2.95-4.53 U/L.

In patients aged 35–37 years, endometrial thickness was positively associated with CPR (adjusted OR = 1.166, 95% CI: 1.025-1.326, P = 0.019). In patients aged ≥38 years, no significant association was observed between basal LH and CPR (P = 0.066).

### 3.5 Multivariate logistic regression analysis of factors influencing live birth rates following fresh embryo transfer in patients of different ages

**Table 6** presents the multivariable logistic regression analysis for LBR. In patients aged <35 years, basal LH was significantly associated with LBR (P = 0.030). Compared with the <25th group, the >75th group had significantly higher LBR (adjusted OR = 1.702, 95% CI: 1.146-2.529, P = 0.008). Other variables were not significantly associated with LBR in this age group.

**Table 6:** Multifactorial logistic regression analysis of live births after fresh embryo transfer in patients of different ages.

Item	<35 years old		35–37 years old		≥38 years old	
	Adjusted OR (*95%CI*)	*P*	Adjusted OR (*95%CI*)	*P*	Adjusted OR (*95%CI*)	*P*
Age	0.987 (0.939~1.037)	0.608	1.015 (0.615~1.675)	0.954	0.817 (0.585~1.139)	0.233
BMI≥24 kg/m^2^	1.110 (0.806~1.528)	0.523	2.306 (0.913~5.821)	0.077	2.120 (0.707~6.354)	0.180
Infertility factors		0.769		0.745		0.527
Tubal factors	Reference		Reference		Reference	
Male factors	0.875 (0.631~1.215)	0.426	1.113 (0.453~2.733)	0.816	1.625 (0.333~7.919)	0.548
Unexplained causes	0.893 (0.587~1.360)	0.598	0.602 (0.125~2.888)	0.526	0.664 (0.039~11.205)	0.777
Others	1.090 (0.690~1.721)	0.712	0.549 (0.126~2.392)	0.424	2.297 (0.689~7.653)	0.176
AFC	0.999 (0.976~1.023)	0.941	1.019 (0.947~1.096)	0.621	1.054 (0.927~1.198)	0.420
Basal AMH	1.041 (0.946~1.146)	0.409	0.829 (0.606~1.135)	0.242	0.947 (0.571~1.568)	0.831
Basal LH		0.030		0.633		0.439
<25*th*	Reference		Reference		Reference	
25*th*–75*th*	1.274 (0.907~1.790)	0.162	0.656 (0.236~1.824)	0.420	0.449 (0.125~1.608)	0.219
>75*th*	1.702 (1.146~2.529)	0.008	0.960 (0.306~3.013)	0.944	0.834 (0.188~3.698)	0.812
Treatment protocol		0.628		0.043		0.162
Antagonist protocol	Reference		Reference		Reference	
Long protocol	1.199 (0.828~1.736)	0.338	1.163 (0.389~3.482)	0.787	3.384 (0.956~11.973)	0.059
Ultra-long protocol	1.136 (0.781~1.651)	0.506	3.451 (1.154~10.321)	0.027	1.798 (0.340~9.510)	0.490
P on hCG injection day	0.671 (0.446~1.010)	0.056	1.496 (0.493~4.541)	0.477	0.867 (0.147~5.117)	0.875
Endometrial thickness on embryo transfer day	1.003 (0.952~1.056)	0.918	1.214 (1.056~1.394)	0.006	1.223 (0.936~1.599)	0.140
Number of high-quality embryos	1.014 (0.965~1.065)	0.576	0.987 (0.833~1.169)	0.879	1.104 (0.885~1.377)	0.382

The interquartile range (25th–75th) for LH in patients<35 years of age was 2.79-4.96 U/L; in patients 35–37 years of age,2.90-4.77 U/L; and in patients ≥38 years of age,2.95-4.53 U/L.

In patients aged 35–37 years, endometrial thickness (adjusted OR = 1.214, 95% CI: 1.056-1.394, P = 0.006) and Treatment protocol(P = 0.043) were associated with LBR. In patients aged ≥38 years, no significant association was observed between basal LH and LBR.

## 4 Discussion

This study investigated the relationship between basal LH levels and pregnancy outcomes following fresh embryo transfer in IVF/ICSI cycles across different age groups. In patients aged <35 years, elevated basal LH levels were associated with higher clinical pregnancy rate and live birth rate, whereas no significant association was observed in the 35–37 years or ≥38 years groups. A progressive decline in HCG positivity rate, clinical pregnancy rate, implantation rate, and live birth rate with increasing age was also observed.

The concept of an LH therapeutic window has been proposed to describe the optimal range of LH concentrations required for successful follicular development and oocyte maturation during COH (9). Excessively low LH levels may lead to insufficient estrogen, impairing follicular development and maturation (10); conversely, excessively high LH levels may promote granulosa cell apoptosis through cytotoxic cytokine effects, resulting in the atresia of immature follicles (11–13). Our data indicate that this window may be particularly relevant in younger women. In patients aged <35 years, after adjusting for confounders, basal LH levels in the highest quartile were significantly associated with higher CPR and LBR compared with the lowest quartile. This finding is similar to previous reports by Noci et al. (14) who observed that low LH levels on day 3 indicate a reduced response to ovarian stimulation. Another study found that patients with low basal LH levels (≤3 U/L) did not exhibit a distinct ovarian response during ovulation induction but had poorer pregnancy outcomes compared to those with LH ≥3 U/L (15). This pattern is consistent with recent data from Zhao et al. (16) who reported in a retrospective analysis of 217 IVF/ICSI cycles that trigger-day LH levels within an optimal range were associated with substantially higher pregnancy rates among women aged ≤35 years, whereas no such protective effect was observed in older patients.

In women aged ≥35 years, the absence of a protective association with elevated LH may be attributable to a series of age-related biological changes in the ovary. First, the primordial and growing follicle pool undergoes accelerated depletion, leaving fewer follicles capable of responding to gonadotropin stimulation (17). Second, the oocytes that remain are progressively compromised by cumulative oxidative damage and declining mitochondrial function, which erode their developmental competence (18, 19). Third, granulosa cells surrounding developing follicles downregulate LH/choriogonadotropin receptor (LHCGR) expression, weakening the follicular response to circulating LH and effectively disconnecting intrafollicular steroidogenesis from systemic LH levels (20). Fourth, the intraovarian endocrine environment shifts with age, including a tendency toward higher late-follicular progesterone output, which may further interfere with the physiological actions of LH on both follicle maturation and endometrial preparation (21).

Beyond the role of LH, several other factors showed associations with pregnancy outcomes. In the <35 years group, progesterone level on the day of hCG administration was negatively associated with clinical pregnancy rate. This is consistent with previous studies reporting reduced clinical pregnancy rates in fresh embryo transfer cycles when late-follicular phase progesterone is elevated (22). The underlying mechanism may involve premature secretory transformation of the endometrium and altered gene expression profiles during the implantation window, which could impair endometrial receptivity (23–25).

In the 35–37 years group, greater endometrial thickness on the day of embryo transfer may be associated with higher clinical pregnancy rate and live birth rate. This finding is consistent with multiple studies documenting that a thin endometrium is associated with reduced implantation and clinical pregnancy rates (26). The prominence of this factor in the 35–37 years age group may be explained by the fact that ovarian function has begun to decline, while uterine aging may not yet be advanced (27). Endometrial receptivity may therefore play a particularly important role during this transitional reproductive stage. Additionally, treatment protocol was significantly associated with live birth rate in this age group, with the ultra-long protocol showing a higher live birth rate compared with the antagonist protocol (28, 29). This may be explained by the earlier and more profound pituitary suppression achieved with the ultra-long protocol, which may help prevent premature LH surges and subtle progesterone elevations during the follicular phase (30), thereby benefiting follicular synchrony and endometrial development. However, given the retrospective and non-randomized nature of protocol assignment, this association should be interpreted cautiously and requires confirmation in future prospective studies.

BMI was also associated with reduced live birth rate specifically in the 35–37 years group. This observation parallels recent findings that the detrimental effect of elevated BMI on IVF outcomes is more evident in younger women and attenuates with advancing age (31, 32). One possible explanation for this pattern is a dynamic interaction between metabolic and ovarian factors. In younger women with favorable ovarian reserve, the adverse metabolic effects of elevated BMI may be partially compensated for (32, 33). In women aged ≥38 years, the growing influence of reproductive aging on oocyte quantity and quality may reduce the relative contribution of BMI to pregnancy outcomes (33–35).

In the ≥38 years group, older age was associated with lower clinical pregnancy rate. This is consistent with previous studies documenting that clinical pregnancy rates decline progressively beyond 38 years of age, reflecting accelerated decreases in both oocyte quantity and quality (8). In the ≥38 years subgroup, although our data do not support a significant association between basal LH and pregnancy outcomes, the confidence intervals for basal LH were notably wide. This imprecision may reflect the limited sample size combined with greater heterogeneity in ovarian aging and endocrine profiles within this age group (16). Consequently, the possibility of a clinically meaningful association cannot be ruled out. Dedicated studies with larger samples of women of advanced reproductive age are needed to clarify this relationship.

This study aimed to investigate the relationship between basal LH levels and pregnancy outcomes following fresh embryo transfer in IVF/ICSI across different age groups. Rather than using a single LH threshold to distinguish low-LH from high-LH groups, quartile intervals were employed for stratification. This approach avoids statistical discrepancies arising from inappropriate LH level categorization.

We acknowledge several limitations inherent to the retrospective, single-center design of this study. First, despite our stringent inclusion and exclusion criteria, selection bias may still exist. This may limit the generalizability of our findings to the broader IVF population and could potentially overestimate the prognostic value of basal LH. Furthermore, the single-center nature of the analysis means that our results may be influenced by center-specific clinical practices and patient demographics, further limiting the generalizability of our conclusions. Additionally, information bias cannot be entirely ruled out, as the retrospective data collection process is inherently susceptible to incomplete or inconsistent documentation. Therefore, further prospective, multicentre studies with larger sample sizes are needed to validate these findings.

In summary, basal serum LH levels in patients aged <35 years are positively associated with clinical pregnancy rate and live birth rate after fresh embryo transfer, while this association is not observed in women aged ≥35 years. Further prospective studies are warranted to validate these findings.

## Data Availability

The original contributions presented in the study are included in the article/**Supplementary Material**. Further inquiries can be directed to the corresponding author.
