# Supplementary material for: Effect of basal LH levels on pregnancy outcome after IVF/ICSI fresh embryo transfer in patients of different ages: a retrospective study
Source: Front Endocrinol (Lausanne). 2026 May 15;17:1820385. doi: 10.3389/fendo.2026.1820385 (PMC13218916; doi:10.3389/fendo.2026.1820385)
Supplement: Supplementary file 1 [file Table1.docx]

Supplementary Table 1 Univariate logistic regression analysis for clinical pregnancy rate in patients of different age groups

| Item | <35 years old | | 35-37 years old | | ≥38 years old | |
| --- | --- | --- | --- | --- | --- | --- |
|  | OR | P | OR | P | OR | P |
| Age | 0.986 | 0.573 | 1.151 | 0.489 | 0.722 | 0.007 |
| BMI | 1.000 | 0.985 | 1.219 | 0.001 | 1.006 | 0.926 |
| AFC | 1.010 | 0.289 | 1.015 | 0.583 | 1.117 | 0.004 |
| Infertility factors |  | 0.810 |  | 0.878 |  | 0.845 |
| Tubal factors | Reference |  | Reference |  | Reference |  |
| Male factors | 0.863 | 0.365 | 0.890 | 0.761 | 1.454 | 0.531 |
| Unexplained causes | 0.924 | 0.705 | 0.837 | 0.777 | 2.077 | 0.489 |
| Others | 0.878 | 0.563 | 0.639 | 0.422 | 1.315 | 0.540 |
| Basal E_2_ | 0.996 | 0.205 | 0.992 | 0.221 | 0.995 | 0.644 |
| Basal P | 1.032 | 0.647 | 1.147 | 0.270 | 1.082 | 0.616 |
| Basal FSH | 1.012 | 0.715 | 0.946 | 0.411 | 0.936 | 0.366 |
| Basal LH |  | 0.003 |  | 0.313 |  | 0.651 |
| <25th | Reference |  | Reference |  | Reference |  |
| 25th-75th | 1.438 | 0.027 | 0.548 | 0.136 | 0.873 | 0.781 |
| >75th | 1.912 | 0.001 | 0.765 | 0.563 | 1.360 | 0.580 |
| Basal AMH | 1.076 | 0.060 | 1.003 | 0.978 | 1.411 | 0.016 |
| Dosage of Gn used | 0.999 | 0.353 | 1.003 | 0.110 | 0.999 | 0.955 |
| Ovulation protocol |  | 0.228 |  | 0.154 |  | 0.043 |
| Antagonist protocol | Reference |  | Reference |  | Reference |  |
| Long protocol | 1.234 | 0.243 | 1.312 | 0.518 | 2.523 | 0.044 |
| ultra-long protocol | 1.352 | 0.086 | 2.187 | 0.060 | 3.392 | 0.026 |
| E_2_ on hCG injection day | 0.999 | 0.655 | 0.999 | 0.502 | 1.001 | 0.217 |
| LH on hCG injection day | 0.646 | 0.028 | 0.810 | 0.656 | 0.810 | 0.729 |
| P on hCG injection day | 0.948 | 0.156 | 0.918 | 0.347 | 0.855 | 0.144 |
| Endometrial thickness | 1.022 | 0.406 | 1.130 | 0.035 | 1.093 | 0.306 |
| Number of high-quality embryos | 1.027 | 0.270 | 1.127 | 0.093 | 1.146 | 0.092 |
| embryo transfer stage (cleavage stage vs. blastocyst stage) | 0.953 | 0.723 | 1.008 | 0.983 | 1.867 | 0.156 |

Supplementary Table 2 Collinearity diagnostics for variables included in the multivariable logistic regression model

| Variable | Tolerance | VIF |
| --- | --- | --- |
| Age | 0.910 | 1.099 |
| AFC | 0.654 | 1.530 |
| Basal AMH | 0.650 | 1.538 |
| Basal LH | 0.959 | 1.043 |
| P on hCG injection day | 0.955 | 1.047 |
| Endometrial thickness on embryo transfer day | 0.978 | 1.022 |
| Number of high-quality embryos | 0.909 | 1.100 |

Supplementary Table 3 Comparison of clinical characteristics of basal LH stratification in the <35 age group [M(IQR),%]

| Item | <35 years old | | | *Z*/$\boldsymbol{x}^{\boldsymbol{2}}$ value | *P* value |
| --- | --- | --- | --- | --- | --- |
|  | <25th | 25th-75th | >75th |  |  |
| No. of cases | 223 | 449 | 222 |  |  |
| Age | 30(4) | 31(4.0) | 30(4.0) | 3.72 | 0.156 |
| BMI (kg/m^2^) | 22.8(3.6) | 21.5(3.8) | 20.7(3.5) | 45.45 | <0.001 |
| Basal E_2_ (pg/mL) | 40.1(22.2) | 41.1(21.1) | 41.8(20.2) | 3.40 | 0.183 |
| Basal LH (U/L) | 2.2(0.9) | 3.8(1.1) | 6.0(1.5) | 752.34 | <0.001 |
| Basal FSH (U/L) | 6.9(2.3) | 7.7(2.2) | 8.4(2.6) | 85.93 | <0.001 |
| Basal AMH (ng/mL) | 2.4(2.0) | 2.7(1.9) | 3.4(2.6) | 37.85 | <0.001 |
| AFC | 14(7) | 15(8) | 17(9) | 18.59 | <0.001 |
| Type of infertility (%) |  |  |  | 3.95 | 0.139 |
| Primary infertility | 54.7(122/223) | 59.5(267/449) | 64.0(142/222) |  |  |
| Secondary infertility | 45.3(101/223) | 40.5(182/449) | 36.0(80/222) |  |  |
| E_2_ on hCG injection day (pg/mL) | 2397.7(1496.0) | 2616.0(1348.1) | 2756.1(1684.5) | 1.41 | 0.012 |
| LH on hCG injection day (U/L) | 1.0(1.1) | 1.1(1.3) | 1.1(1.4) | 8.87 | 0.262 |
| P on hCG injection day (ng/mL) | 0.9(0.5) | 0.9(0.4) | 0.9(0.5) | 0.89 | 0.620 |
| Endometrial thickness (mm) | 12.0（3.7） | 11.9(2.9) | 12.0(3.7) | 2.20 | 0.333 |
| Number of embryos transferred | 2(1) | 2(1) | 2(1) | 0.96 | 0.494 |

Note: The interquartile range (25th-75th) for LH in patients <35 years of age was 2.79-4.96 U/L.

Supplementary Table 4 Comparison of clinical characteristics of basal LH stratification in the 35-37 age group [M(IQR),%]

| Item | 35-37 years old | | | *Z*/$\boldsymbol{x}^{\boldsymbol{2}}$ value | *P* value |
| --- | --- | --- | --- | --- | --- |
|  | <25th | 25th-75th | >75th |  |  |
| No. of cases | 38 | 74 | 37 |  |  |
| Age | 36(1) | 36(2) | 36(1) | 0.45 | 0.798 |
| BMI (kg/m^2^) | 23.8(4) | 21.8(3.1) | 20.8(3.8) | 20.95 | <0.001 |
| Basal E_2_ (pg/mL) | 41.2 (18.0) | 44.2(20.8) | 45.0(26.1) | 0.91 | 0.636 |
| Basal LH (U/L) | 2.2(1.0) | 3.8(0.8) | 5.8(1.6) | 125.16 | <0.001 |
| Basal FSH (U/L) | 7.4(2.8) | 8.0(2.7) | 9.2(2.7) | 11.66 | 0.003 |
| Basal AMH (ng/mL) | 2.0(1.7) | 2.1(1.5) | 2.4(1.8) | 1.73 | 0.420 |
| AFC | 13(9) | 12.5(7) | 14(11) | 1.35 | 0.508 |
| Type of infertility (%) |  |  |  | 0.14 | 0.933 |
| Primary infertility | 44.7(17/38) | 43.2(32/74) | 40.5(15/37) |  |  |
| Secondary infertility | 55.3(21/38) | 56.8(42/74) | 59.5(22/37) |  |  |
| E_2_ on hCG injection day (pg/mL) | 1908.5 (1736.5) | 2329.8(1499.3) | 2493.9(1513.2) | 2.71 | 0.258 |
| LH on hCG injection day (U/L) | 1.1(1.7) | 1.2(1.7) | 1.6(2.3) | 2.00 | 0.368 |
| P on hCG injection day (ng/mL) | 0.9 (0.6) | 0.9(0.4) | 0.9(0.4) | 0.40 | 0.818 |
| Endometrial thickness (mm) | 11.6(2.8) | 12.0(3.3) | 11.2(4.2) | 2.08 | 0.353 |
| Number of embryos transferred | 2(0.25) | 2(1) | 2(1) | 1.92 | 0.383 |

Note: The interquartile range (25th-75th) for LH in patients 35-37 years of age was 2.90-4.77 U/L.

Supplementary Table 5 Comparison of clinical characteristics of basal LH stratification in the ≥38 age group [M(IQR),%]

| Item | ≥38 years old | | | *Z*/$\boldsymbol{x}^{\boldsymbol{2}}$ value | *P* value |
| --- | --- | --- | --- | --- | --- |
|  | <25th | 25th-75th | >75th |  |  |
| No. of cases | 27 | 56 | 27 |  |  |
| Age | 39(4) | 39(2) | 40(3) | 3.03 | 0.220 |
| BMI (kg/m^2^) | 22.1(4.5) | 22.8(3.2) | 20.8(4.7) | 3.77 | 0.152 |
| Basal E_2_ (pg/mL) | 36.0(25.8) | 48.6 (24.2) | 47.9(13.6) | 6.76 | 0.034 |
| Basal LH (U/L) | 2.1(1.1) | 3.8(1.1) | 5.5(2.2) | 91.41 | <0.001 |
| Basal FSH (U/L) | 7.2(3.0) | 8.2 (2.4) | 9.2(4.3) | 7.74 | 0.021 |
| Basal AMH (ng/mL) | 1.9(1.5) | 1.8 (1.6) | 1.5 (1.4) | 1.33 | 0.515 |
| AFC | 9(8) | 12(6.0) | 9(7) | 4.16 | 0.125 |
| Type of infertility (%) |  |  |  | 0.93 | 0.630 |
| Primary infertility | 33.3(9/27) | 25.0(14/56) | 33.3(9/27) |  |  |
| Secondary infertility | 66.7(18/27) | 75.0(42/56) | 66.7(18/27) |  |  |
| E_2_ on hCG injection day (pg/mL) | 2360.8(1666.6) | 2220.2(1679.0) | 2209.5(1954.0) | 0.42 | 0.812 |
| LH on hCG injection day (U/L) | 1.9(2.2) | 1.6(2.3) | 1.8(3.2) | 3.52 | 0.172 |
| P on hCG injection day (ng/mL) | 0.8(0.4) | 0.8 (0.4) | 0.9(0.4) | 0.42 | 0.813 |
| Endometrial thickness (mm) | 10.2(2.5) | 11.3(2.7) | 9.7(2.9) | 13.33 | 0.001 |
| Number of embryos transferred | 2(1) | 2(0) | 2(1) | 0.64 | 0.727 |

Note: ≥38 years of age, The interquartile range (25th-75th) for LH was 2.95-4.53 U/L.
